# Youth characteristics in relation to their perceptions of sexual consent: a cross-sectional study

**DOI:** 10.1136/bmjpo-2025-004157

**Published:** 2026-06-22

**Authors:** Cyril Knob, Lorraine Chok, Romaine Delacrétaz, Yara Barrense-Dias, Anne- Emmanuelle Ambresin

**Affiliations:** 1 University of Lausanne, Lausanne, Switzerland; 2 Interdisciplinary Division for Adolescent Health, Lausanne University Hospital Department of Woman Mother and Child, Lausanne, Switzerland; 3 Department of Epidemiology and health system, Unisanté, Lausanne, Switzerland

**Keywords:** Adolescent Health, Health Policy, Cross-Sectional Studies

## Abstract

**Background:**

Ensuring sexual health and preventing sexual violence among adolescents hinges on a comprehensive understanding of sexual consent.

**Objective:**

The purpose of this study is to explore the individual, family, health and social factors associated with the perceptions and understanding of sexual consent among young people in Switzerland.

**Methods:**

Data were drawn from a national cross-sectional survey on adolescent mental health conducted between June and August 2021 in Switzerland (N=988; ages 14–19). Participants completed a self-reported online questionnaire disseminated via social media. The survey included seven items assessing perceptions of sexual consent. Based on responses, participants were classified into three groups according to their perception of consent: ‘affirmative’, ‘hybrid’ and ‘implicit’. Associations with sociodemographic characteristics, health status, risky behaviour, exposure to violence and healthcare utilisation were examined using bivariate analyses and multinomial logistic regressions.

**Results:**

Findings indicate that up to 15.8% of participants held ‘implicit’ perceptions of sexual consent. Gender differences emerged, particularly in the definitions of rape and coercion. Multivariable analyses indicated that males (relative risk ratio, RRR=2.21), younger adolescents aged 14–15 (RRR=3.00), those with below-average perceived socioeconomic status (RRR=2.86) and youth with non-standard occupation (RRR=4.57) were more likely to fall into the ‘implicit’ perception group.

**Conclusions:**

This study offers valuable insights to better understand adolescents’ perceptions of sexual consent, which is essential for designing developmentally appropriate prevention and education strategies that support healthy sexual development—one of the key developmental tasks of this life stage.

**WHAT IS ALREADY KNOWN ON THIS TOPIC**

- Adolescence is a critical developmental stage marked by emerging sexual and relational experiences, during which understanding and communicating sexual consent is essential for promoting sexual health and preventing sexual violence.

**WHAT THIS STUDY ADDS**

- Drawing on a large and diverse adolescent sample, this study identifies persistent complexities and disparities in perceptions of sexual consent across gender, age, socioeconomic status, educational pathways and behavioural factors, underscoring the need for targeted, developmentally appropriate consent education and healthcare engagement.

**HOW THIS STUDY MIGHT AFFECT RESEARCH, PRACTICE OR POLICY**

- These findings reinforce the importance of integrating sexual consent education into adolescent healthcare, tailored to developmental stages and social contexts. The study offers timely evidence to inform public health, educational practice and policy during a period of international legislative reform on sexual consent.

## Introduction

 Adolescence represents a fundamental stage of development, where the exploration of sexual and gender identity, attractions, relationships, dating and intimacy is central.^1^ This exploration is particularly significant, as many adolescents begin to engage in their first romantic and/or sexual experiences.^2^ Consequently, this period is crucial for strengthening education and prevention measures against relationship abuse and sexual violence.^3^ Indeed, the onset of sexual activity during adolescence is often associated with an increased risk of sexual violence.^4^

Sexual assault is intricately linked to the notion of sexual consent.^5^ Ensuring sexual health and preventing sexual violence hinges on a comprehensive understanding of sexual consent.^6^ However, the definition and operationalisation of sexual consent remain conceptually diverse. Three broad conceptual approaches can be distinguished: (1) implicit consent models, in which consent is assumed absent explicit refusal^7 8^; (2) affirmative models, in which consent is defined as a clear, voluntary and mutual actively expressed agreement^8 9^ and (3) contextual/relational models, in which consent is negotiated through non-verbal cues and relationship history.^6 7^ While affirmative consent (‘yes-means-yes’ approach) has become the standard for many sex education curricula,^10 11^ its implementation is consistently shown to be uneven.

Previous studies have shown that adolescents generally support affirmative consent practices, although the conceptualisation of sexual consent can be complex and take different forms.^12 13^ While some research indicates that adolescents define sexual consent primarily through verbal communication,^14 15^ real-life encounters frequently rely on non-verbal cues and indirect signals,^7 16^ with females being more likely than males to use verbal strategies, and males, particularly younger ones, more readily interpreting ambiguity or the absence of refusal as tacit consent,^6 15 17 18^ a pattern that tends to attenuate with age.^19^ These differences are further shaped by contextual factors such as relationship intimacy, prior sexual experiences and substance use.^20 21^ Underlying these variations are deeper social, cultural and psychological barriers to the enactment of affirmative consent: gendered sexual scripts, power asymmetries, socioeconomic status and sexual or emotional minority identity, all constrain the ability to give or withhold consent freely—particularly for women and young people from sexual and gender minorities.^8 12 14^

These representations of consent and the barriers shaping them are not context-free: social, legal, cultural and educational factors play a decisive role in how young people perceive and negotiate sexual consent.^8^ This study echoes recent Swiss legal reforms (July 2024), which replaced a coercion-based definition of rape with a broader ‘no-means-no’ framework, recognising verbal, gestural and non-responses such as state of shock as valid forms of refusal.^22^ This legislative shift in Switzerland mirrors a broader international movement towards strengthening legal definitions of sexual consent.

Promoting positive sexuality and comprehensive sex education, with a focus on respectful relationships and effective communication, has been identified as a protective factor against both experiencing and perpetrating non-consensual sexual acts.^23^ This, combined with the fact that affirmative consent communication between partners is linked to healthier relations,^24^ supports the promotion of and equal access to accurate sex education including sexual consent with active involvement of healthcare providers. However, the literature shows that this has not yet been achieved.^25 26^

The purpose of this study is to explore youth perceptions and understanding of sexual consent in relation to their characteristics, including demographic, health and social factors, among young people in Switzerland. We hypothesise that (1) distinct clusters of adolescents could be identified based on their positioning on sexual consent and (2) these clusters may be associated with their sociodemographic and health profiles.

## Methods

### Sample

**Figure 1:**
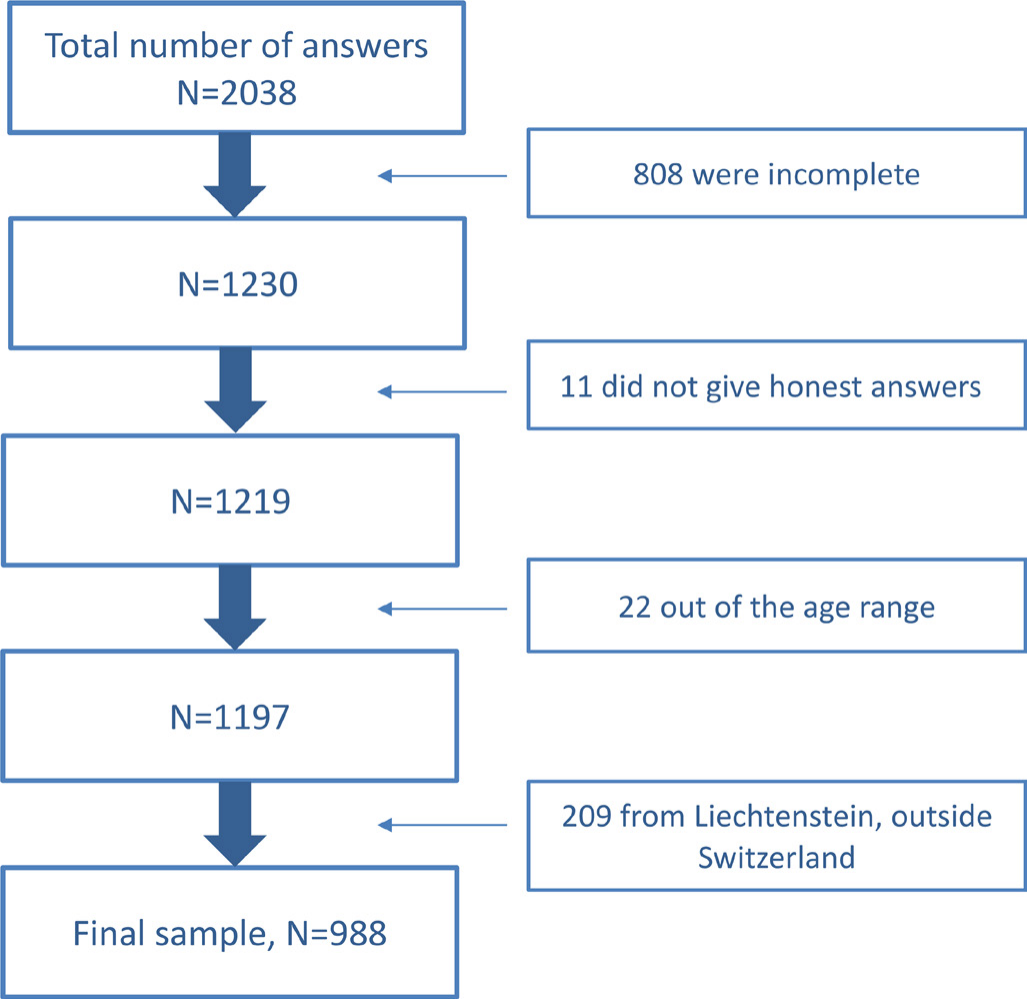
Participants’ flow chart.

Data were obtained from a national cross-sectional survey on the mental health of young people aged 14–19 years in Switzerland and Liechtenstein in which participants responded to questions about sexual consent (secondary analysis of data from a research study originally designed to assess the prevalence of mental health disorders (depression and/or anxiety) among adolescents).^27^ Data were collected through an online self-reported questionnaire disseminated through social media between June and August 2021. Participation was voluntary and the questionnaire was available in the official languages of both countries. Responses were collected in a manner that ensured individuals could not be identified. Participants who indicated that their answers were not honest enough to be used for research purposes were excluded.^28 29^ The final sample (N=988, figure 1) is weighted according to age, assigned sex at birth and region of residence.

### Measures

Participants were presented with eight statements on sexual consent and had to answer whether they agreed, disagreed or did not know. Answers were dichotomised into those corresponding to affirmative and active consent versus those that did not correspond (including ‘did not know’ answers).^10 11^

Various independent variables that could have a relation with the perceptions of sexual consent were considered to explore the characteristics of the participants. Sociodemographic and personal data were used including age, gender (male, female, other), region of residence (German-speaking, French-speaking or Italian-speaking Switzerland), perceived socioeconomic status based on the ESPAD survey^30^ (above average, average, below average compared with other families in Switzerland), occupation (mandatory education, post-mandatory education, apprenticeship/work, non-standard occupation defined as no activity, job-seeking or any other situation outside school, training or employment) and family structure (parents together, other situation).

Substance use (tobacco, alcohol misuse/drunkenness, cannabis and other drugs) and antisocial behaviours (shoplifted, participated in a fight, destroyed someone else’s property, used transportation without a valid ticket) were analysed as dichotomous variables, categorised as yes (at least once in the last 30 days) and no.

Having experienced physical or verbal violence from a partner (yes/no) and having been a victim of sexual abuse (yes/no) were also included as independent variables.

Emotional health data included lifetime suicidal thoughts and attempts categorised as yes, no, I don’t want to answer, considering only positive responses and combining *n*o and I don’t want to answer, self-esteem using the Rosenberg Self-Esteem Scale^31^ and anxiety assessed with the Generalised Anxiety Disorder Questionnaire-7^32^ scoring in four categories, minimal anxiety, mild anxiety, moderate anxiety and severe anxiety. Participants were also asked about their main resource for emotional difficulties and possible responses were combined into family (including parents, siblings and other family members), peers (friends and girl/boyfriends), health professional, school professional, no resource and others. Responses were then dichotomised into at least one resource and no resource.

Perceived somatic health was assessed using a five-point scale ranging from excellent to poor and responses were dichotomised as good including excellent, very good and good, and poor including poor and very poor.

Healthcare use was evaluated based on whether participants had visited a mental health professional or general practitioner at least once in the past 12 months. Responses were considered negative if the participant had not had a visit during that time or if the participant could not recall.

### Analysis

The eight statements on sexual consent were developed by the authors, drawing on the principles underlying ongoing Swiss legal reforms^22^ and the sexual consent literature,^8^,^10^ according to which affirmative consent must be actively expressed, freely given, clear and cannot be inferred from silence, passivity or contextual cues. The statements were designed to capture a range of consent representations from affirmative to contextual or presumptive approaches. After an initial analysis (data not shown), one statement (‘It is not always easy to know when a person wants to have sex or not’) was removed due to the risk of measurement bias, as agreement could reflect either an awareness of the importance of explicit consent or a tolerance for ambiguity in sexual situations, making it difficult to assign a consistent direction.

For the remaining seven statements (table 1), responses were coded by the authors as consistent (scored 0) or inconsistent (scored 1) with the affirmative sexual consent standards. Specifically, disagreement with statement 1 and agreement with statements 2–7 were coded as inconsistent with this standard. Based on the overall distribution of scores, participants were classified into three groups reflecting a continuum of sexual consent perceptions: (1) ‘Affirmative perception’—score of 0, all responses consistent with affirmative consent; (2) ‘Hybrid perception’—score of 1–2, reflecting partial alignment with affirmative consent norms while retaining some reliance on contextual or presumptive cues and (3) ‘Implicit perception’—score of 3 or more, reflecting a presumptive or non-refusal consent model. The cut-off of 3 or more for the implicit perception group was determined based on the overall distribution of scores, reflecting a pattern of responses predominantly inconsistent with affirmative consent principles across the majority of statements.

**Table 1:** Level of agreement to situations displaying sexual consent by gender

	Total (N=988)	Male (N=483)	Female (N=477)	Other (N=28)	P value
1. To have any kind of sexual relationship, your partner must give explicit consent (must clearly say Yes)	87.0%	85.5%	88.3%	92.6%	0.464
2. If a person allows to be kissed easily, it is a sign that s/he wants to have sex	9.7%	12.7%	6.5%	11.1%	0.030*
3. If a person dresses in a provocative/sexy way, it is a sign that s/he wants to have sex	10.0%	14.5%	5.7%	2.1%	0.003**
4. If a person doesn't say ‘no’, it means that s/he wants to have sex	8.7%	10.1%	7.3%	7.4%	0.401
5. If a person does not want to have sex, s/he must say so clearly	59.9%	64.8%	55.1%	55.6%	0.079
6. To be considered rape, it must involve some form of violence or threats by the author	21.9%	28.2%	16.1%	14.8%	0.001**
7. To be considered rape, the victim must fight to defend her/himself	13.2%	16.5%	10.4%	3.7%	0.042*

*p<0.05, **p<0.01.

All percentages reflect weighted sample estimates. χ² tests were used for all comparisons.

To ensure representativeness of the sample with respect to key demographic variables, statistical weights were calculated. These weights were constructed based on official statistics so that the tri-variate distribution of biological sex (female/male), age and region of residence in the sample would match that of the target population—young people aged 14–19 residing in Switzerland. While the sample was not drawn using probabilistic sampling methods, the use of poststratification weights helps to correct for potential imbalances and improve the generalisability of the findings. Each age from 14 to 19 years was considered separately. Participants who answered ‘other’ were assigned a unit weight because their actual proportion in the Swiss population is not currently officially known. All subsequent statistical analyses were conducted using these weights. No missing data were identified, as only complete questionnaires were retained.

First, bivariate analyses were conducted to determine means and prevalence rates. χ² tests for categorical variables and analysis of variance for continuous ones were used to compare the three groups. Subsequently, a multinomial logistic regression was performed to examine the association between factors identified as being significant at the bivariate level with the three groups, using the ‘affirmative perception’ group as the reference category. Furthermore, subgroup and interaction analyses were conducted, with gender used as a stratification factor. Statistical significance was set at a level of 0.05.

Stata V.18 (Stata) was used for all computations.

### Patient and public involvement

The study’s design did not involve patients or the general public. However, all participating patients were informed of the research objectives and their informed consent was obtained. The survey was completed by participants voluntarily and no input from patients was sought in interpreting or writing up the results. The results of the research will not be disseminated to the patients.

## Results

Of the 988 participants of the final sample, 48.9% identified as male, 48.3% as female and 2.8% defined their gender as other. Participants’ characteristics are reported in table 2.

**Table 2:** Participants’ characteristics. Young people aged 14–19 years in Switzerland

	All young people	
	Weighted sample (N)	Weighted sample (%)
Overall	988	100
Age (mean±SD)	988	16.52±0.09
14–15 years old 16–17 years old 18–19 years old	322 331 335	32.6 33.5 33.9
Gender		
Male Female Other	483 477 28	48.9 48.3 2.8
Residence		
German-speaking Switzerland French-speaking Switzerland Italian-speaking Switzerland	622 327 39	63 33.1 3.9
Perceived socioeconomic status		
Below average	108	10.9
Parental situation		
Non intact	273	27.7
Occupation		
Mandatory school Post-mandatory education/university Apprenticeship/work Non-standard occupation/without activity	198 460 273 58	20.0 46.6 27.6 5.8
Substance use (last 30 days)		
Tobacco Alcohol misuse Cannabis and other drugs	364 379 200	36.8 38.3 20.3
Antisocial behaviours (last 30 days)		
Shoplifted Participated in a fight Destroyed someone else’s property Used transportation without a valid ticket	83 104 100 340	8.4 10.5 10.1 34.4
Victim of sexual assault		
Yes	104	10.5
Physical or verbal violence from partner		
Yes	92	9.4
Suicidal thoughts		
Yes	445	45.0
Suicide attempt		
Yes	86	8.7
Self-esteem		
Low self-esteem	312	31.6
Anxiety (GAD-7)		
Minimal Mild Moderate Severe	440 313 147 97	43.6 31.7 14.9 9.8
Emotional support		
At least one Nobody	705 283	71.4 28.6
Somatic health		
Poor	171	17.3
Health professional (this past year)		
Mental health Primary physician	240 620	24.3 62.9

For binary variables, only one category is reported.

GAD-7, 7-item Generalised Anxiety Disorder Scale.

Of 988 young people surveyed, 283 (28.7%) had an ‘affirmative’, 549 (55.5%) a ‘hybrid’ and 156 (15.8%) an ‘implicit’ perception of consent. As shown in table 1, there were significant gender differences in interpretation of consent and the associated definition of rape. Males were more likely than females to agree that if a person allows to be kissed easily (males=12.7%, females=6.5%, p=0.03) or if a person dresses in a provocative/sexy way (males=14.5%, females=5.7%, p=0.003) it is a sign that she/he wants to have sex. In the same trend, males were more likely than females to agree that to be considered rape, it must involve some form of violence or threats by the author (males=28.2%, females=16.1%, p=0.001) or that to be considered rape, the victim must fight to defend her/himself (males=16.5%, females=10.4%, p=0.042).

Table 3 reports distributions and bivariate associations between the participants’ characteristics and their perception of consent. Analysed variables show significant differences regarding gender and occupation. Females were more likely to be in the ‘affirmative perception’ group (males=36.2%, females=60.5%, p=0.001), while males were more likely to be in the ‘implicit perception’ group (males=63.5%, females=34.6%, p=0.001). Participants in the ‘affirmative perception’ group were also more often enrolled in post-mandatory education (mandatory education=13.8%, post-mandatory education=59.2%, apprenticeship/work=22.3%, non-standard occupation=4.7%, p=0.029), whereas those in the ‘implicit perception’ group were more likely to be in apprenticeships or employed (mandatory education=26.7%, post-mandatory education=32.3%, apprenticeship/work=33.7%, non-standard occupation=6.3%, p=0.029). Furthermore, antisocial behaviours, suicidal thoughts, having attempted suicide, having been sexually assaulted, self-esteem and anxiety level emerge as variables with significant differences at the bivariate level.

**Table 3:** Bivariate analysis—characteristics of participants depending on their perception of sexual consent

	‘Affirmative perception’ (score=0)	‘Hybrid perception’ (score=1–2)	‘Implicit perception’ (score ≥3)	
	**N=283**	**N=549**	**N=156**	**P value**
Overall	28.7%	55.5%	15.8%	
Age (mean±SE)	16.7±0.2*	16.6±0.1*	16.1±0.2*	0.044
14–15 years old	26.0%	33.0%	43.6%	0.065
16–17 years old	38.3%	30.8%	34.1%	0.065
18–19 years old	35.7%	36.2%	22.3%	0.065
Gender				< 0.001
Male	36.2%**	51.4%**	63.5%**	
Female	60.5%**	45.8%**	34.6%**	
Other	3.2%**	2.8%**	2.0%**	
Residence				0.744
German-speaking CH	64.4%	63.8%	57.9%	
French-speaking CH	32.4%	32.5%	36.5%	
Italian-speaking CH	3.1%	3.8%	5.6%	
Perceived socioeconomic status				
Below average	7.9%	11.4%	14.4%	0.235
Parental situation				
Non intact	25.6%	31.2%	19.1%	0.056
Occupation				0.029
Mandatory education	13.8%*	21.3%*	26.7%*	
Post-mandatory education	59.2%*	44.1%*	32.3%*	
Apprenticeship/work	22.3%*	28.4%*	33.7%*	
Non-standard occupation	4.7%*	6.2%*	6.3%*	
Substance use (last 30 days)				
Tobacco	43.5%	35.2%	30.7%	0.126
Alcohol misuse	41.9%	38.3%	31.9%	0.347
Cannabis and other drugs	23.1%	20.6%	14.1%	0.232
Antisocial behaviours (last 30 days)				
Shoplifted	7.6%	8.2%	10.4%	0.715
Participated in a fight	8.3%	9.4%	18.6%	0.167
Destroyed someone else’s property	7.7%*	9.2%*	18.0%*	0.040
Used transportation without a valid ticket	33.9%	34.7%	33.9%	0.981
Victim of sexual assault				
Yes	14.0%*	10.4%*	4.5%*	0.019
Physical or verbal violence from partner				
Yes	8.7%	9.9%	8.6%	0.415
Suicidal thoughts				
Yes	50.3%**	47.0%**	37.9%**	0.007
Suicide attempt				
Yes	10.8%**	9.5%**	2.1%**	0.009
Self-esteem				
Low self-esteem	38.7%*	30.7%*	22.1%*	0.019
Anxiety (GAD-7)				0.049
Minimal	36.9%*	45.4%*	49.6%*	
Mild	35.5%*	27.9%*	37.7%*	
Moderate	16.1%*	16.7%*	6.4%*	
Severe	11.5%*	10%*	6.3%*	
Emotional support				0.350
At least one	69.1%	74.1%	66.1%	
Nobody	30.9%	25.9%	33.9%	
Somatic health				
Poor	18.8%	17.5%	13.9%	0.618
Health professional (this past year)				
Mental health	27.1%	23.8%	21.0%	0.543
Primary physician	71.0%	60.4%	56.7%	0.063

*p<0.05, **p<0.01.

All percentages reflect weighted sample estimates. χ² tests were used for categorical variables and ANOVA for continuous variables.

ANOVA, analysis of variance; GAD-7, 7-item Generalised Anxiety Disorder Scale.

Table 4 displays the results of multinomial logistic regression analysis, confirming that males had higher odds of having ‘implicit’ perception of sexual consent (relative risk ratio, RRR=2.21, 95% CI (1.20 to 4.07), p=0.011) or a ‘hybrid’ perception of sexual consent (RRR=1.64, 95% CI (1.00 to 2.65), p=0.011) than considering sexual consent to be affirmative. Younger people of 14–15 years old (RRR=3.00, 95% CI (1.03 to 8.73), p=0.044) and those with perceived socioeconomic status below average (RRR=2.86, 95% CI (1.34 to 6.05), p=0.006) were also more likely to have an ‘implicit’ perception. Individuals who were in apprenticeship or working were more likely to have an ‘implicit’ perception (RRR=3.06, 95% CI (1.37 to 6.86), p=0.006) or a ‘hybrid’ perception (RRR=1.63, 95% CI (1.03 to 2.59), p=0.006). Young people with a non-standard occupation also had more chances to have an ‘implicit’ perception (RRR=4.57, 95% CI (1.42 to 14.72), p=0.011). Exploring antisocial behaviours, analyses show that individuals who reported having destroyed someone else’s property in the last 30 days were more likely to have an ’implicit’ perception of sexual consent (RRR=2.48, 95% CI (1.01 to 6.16), p=0.049). Finally, individuals who reported having had suicidal thoughts were less likely to be in the ‘implicit perception’ group (RRR=0.49, 95% CI (0.26 to 0.95), p=0.034).

**Table 4:** Multivariate logistic regression: relative risk ratios (RRR–95% CI) to have a hybrid perception of sexual consent (group 2) or an implicit perception (group 3) versus considering sexual consent to be affirmative (group 1–reference category)

	‘Hybrid perception’ (N=549)	P value	‘Implicit perception’ (N=156)	P value
	Relative risk ratio (95% CI)		Relative risk ratio (95% CI)	
Age (mean±SE)				
14–15 years old	1.13 (0.59 to 2.18)	0.712	3.00 (1.03 to 8.73)*	0.044
16–17 years old	0.78 (0.52 to 1.19)	0.248	1.32 (0.66 to 2.63)	0.431
18–19 years old	1		1	
Gender				
Male	1.64 (1.00 to 2.65)*	0.045	2.21 (1.20 to 4.07)*	0.011
Female	1		1	
Other	1.31 (0.55 to 3.15)	0.545	1.53 (0.38 to 6.13)	0.549
Perceived socioeconomic status				
Below average	1.45 (0.85 to 2.48)	0.168	2.86 (1.34 to 6.05)**	0.006
Parental situation				
Non intact	1.41 (0.94 to 2.10)	0.093	0.84 (0.43 to 1.62)	0.595
Occupation				
Mandatory education	1.65 (0.68 to 4.04)	0.271	1.44 (0.48 to 4.33)	0.512
Post-mandatory education	1		1	
Apprenticeship/work	1.63 (1.03 to 2.59)*	0.039	3.06 (1.37 to 6.86)**	0.006
Non-standard occupation	1.68 (0.80 to 3.51)	0.168	4.57 (1.42 to 14.72)*	0.011
Antisocial behaviours (last 30 days)				
Destroyed someone else’s property	1.08 (0.51 to 2.26)	0.848	2.48 (1.01 to 6.16)*	0.049
Victim of sexual assault				
Yes	0.84 (0.51 to 1.37)	0.483	0.57 (0.23 to 1.42)	0.228
Suicidal thoughts				
Yes	0.97 (0.62 to 1.51)	0.889	0.49 (0.26 to 0.95)*	0.034
Suicide attempt				
Yes	0.98 (0.51 to 1.89)	0.951	0.35 (0.12 to 1.06)	0.064
Self-esteem				
Low self-esteem	0.77 (0.49 to 1.21)	0.254	0.92 (0.47 to 1.79)	0.810
Anxiety (GAD-7)				
Minimal	1		1	
Mild	0.75 (0.44 to 1.25)	0.269	1.14 (0.56 to 2.35)	0.713
Moderate	1.04 (0.58 to 1.85)	0.903	0.46 (0.16 to 1.34)	0.156
Severe	0.93 (0.49 to 1.79)	0.834	0.99 (0.40 to 2.43)	0.985

*p<0.05, **p<0.01.

GAD-7, 7-item Generalised Anxiety Disorder Scale.

Using subgroup and interaction analyses, with gender used as a stratification factor (data not shown), ‘implicit’ perception of sexual consent was significantly correlated with perceived socioeconomic status as below average (RRR=2.53, 95% CI (1.18 to 5.40), p=0.017) and males who were in apprenticeship or working (RRR=6.63, 95% CI (1.60 to 27.56), p=0.009).

## Discussion

This study provides important insights into how adolescents can perceive and understand sexual consent, and about the characteristics that may be associated.

Analysis of the responses to the statements indicates that just above half of participants fall within the category of having 1 to 2 responses that do not conform to the standard of active affirmative consent. This may reflect the recent inclusion of affirmative consent in education curricula and evolving legislation,^11 22^ as well as the limitations of quantitative surveys in capturing the nuances of real-life encounters.^7 12 14 15^

Nevertheless, our findings indicate that 15.8% of respondents hold an implicit perception of consent, consistent with a presumptive or non-refusal model. It is therefore essential to continue developing appropriate, developmentally informed, culturally sensitive and contextually relevant interventions that include this subgroup by considering their specific characteristics to tailor targeted messages.

### Gender differences and societal norms

One of the most notable findings is the gender difference in attitudes toward sexual consent. Males were significantly more likely than females to hold an ‘implicit’ perception of consent, such as interpreting clothing or passive behaviour as indicative of consent. Multivariate analysis confirmed this disparity, revealing that males are over twice as likely as females to adopt implicit interpretations of sexual consent.

These gender-based differences could be understood through societal norms and gendered socialisation processes that shape how young men and women interpret sexual interactions and consent. Previous studies^7 14 17^ suggest that men—especially in heterosexual traditional script—are socialised to view sexual initiative as a demonstration of masculinity and therefore are responsible for advancing the sexual interaction. This may lead them to misinterpret cues, to consider the mere absence of explicit refusal as tacit forms of consent or to ‘keep trying’ despite initial resistance.^7 14 15 17^ In contrast, women—socialised to value communication and the explicit expression of boundaries—are more likely to interpret the absence of affirmative consent as an indicator of discomfort or non-consent.^6 33^

Furthermore, these findings highlight the critical need to include young men in educational discussions about consent and sexual communication. The traditional exclusion of boys from conversations around sexual communication with parents or healthcare providers potentially reinforces misunderstandings and harmful interpretations.^15 25 34^ Promoting a clear, shared understanding of affirmative consent among boys and engaging them in discussions about empathy, boundaries and mutual respect is thus essential to reduce problematic perceptions, prevent risky behaviours and promote positive sexuality.^35^

### Age, socioeconomic and educational status

Consistent with previous literature, the present analysis demonstrated that age plays a pivotal role in the differences among perceptions of sexual consent.^18^ Younger adolescents aged 14–15 were three times more likely to fall into the ‘implicit’ perception category compared with their older peers. This suggests younger adolescents may lack the maturity, experience or education to fully comprehend affirmative consent, highlighting the need for early, developmentally appropriate sex education that grows with their cognitive and emotional development.^36^

Analysis revealed that adolescents from lower socioeconomic backgrounds were substantially more likely to hold ‘implicit’ perceptions of sexual consent—an association that persisted in the interaction analysis. These results align with previous research indicating that limited access to comprehensive sex education and health resources, which is often correlated with socioeconomic status, may hinder young people’s understanding of healthy sexual communication.^26 37^

Educational pathways also emerged as critical determinants of consent perception. Young people engaged in apprenticeships or working, as opposed to those pursuing post-mandatory education, were more likely to belong to the ‘implicit perception’ group. This distinction may reflect differences in exposure to sex education, as those in vocational training or early employment might have less structured access to school-based sexual health programmes.^23^ This is particularly relevant in the Swiss context, where sex education is mandatory only during compulsory schooling (ending around ages 15–16), while provision at the post-compulsory level remains non-mandatory. Marked by linguistic-regional differences (such as professionalised delivery in French-speaking regions versus teacher-led instruction in German-speaking areas),^26^ this organisation may amplify inequalities in sexual consent education, particularly for those who leave school early.

### Emotional health and behaviour characteristics

Interestingly, certain emotional and behavioural factors were associated with how adolescents perceive sexual consent. Sexual violence is influenced by a range of risk and protective factors that can be comprehensively examined using the socioecological model.^38^ Scholars have found that at the individual level, behaviours such as alcohol use and antisocial conduct are frequently observed among adolescents who engage in sexually aggressive behaviour.^38 39^ The present study’s findings corroborate this: young people who had engaged in antisocial behaviours, such as destroying property, were more likely to be part of the ‘implicit perception’ group. This association suggests a potential link between behavioural dysregulation and difficulties in recognising or respecting sexual boundaries. It may also reflect broader issues of impulse control or a disregard for social norms, including those governing interpersonal interactions.

Conversely, adolescents who reported experiencing suicidal thoughts were less likely to belong to the ‘implicit perception’ group. However, this finding requires cautious interpretation. One possible explanation could be that young people who experience emotional distress may develop heightened sensitivity to the emotional states of others, potentially fostering greater empathy and awareness of boundaries which has consistently been identified as a protective factor for sexual violence.^38 39^ Alternatively, this group may include individuals who have themselves been victims of adverse experiences, leading them to develop clearer understandings of what constitutes consent.

### Implications for policy and education

The findings have important implications for both educational programming and public policy. Ongoing legal reforms in several countries present a timely opportunity to align legal standards with comprehensive sex education and public awareness campaigns.^11^ Promoting affirmative consent not only contributes to violence prevention, but also to the broader goal of supporting positive youth development and well-being through positive sexuality.^24^ Therefore, schools, healthcare providers, parents and community organisations must collaborate to deliver consistent and accurate messages about sexual consent.^11 34^ This study also reinforces the need to integrate sexual consent discussions into adolescent mental health and primary care, with providers trained to address relationships, consent and sexual health in a developmentally appropriate way.^40^

### Strength, limitations and future research

A major strength of this study is its large and diverse sample, which was not restricted to school-attending or clinical populations and thus provides valuable insights beyond these often-studied groups. While the data are only weighted to reflect age, gender and region, the inclusion of adolescents from varied socioeconomic and educational backgrounds strengthens its relevance. Importantly, the study captures perceptions of sexual consent at a critical developmental stage (ages 14–19), marked by identity, relational and sexual transitions. Collecting such data during an international period of reflection and legislative reform around sexual consent and rape further strengthens its contribution to a topic that remains insufficiently explored.^40^

Several limitations should be acknowledged. First, the study relies on self-reported data, which may be influenced by social desirability bias or inaccuracies due to memory recall. Second, the use of a self-administered survey raises the possibility of inattentive responding. Third, the study did not specifically investigate verbal versus non-verbal forms of sexual consent communication, nor did it collect data on participants’ sexual activity or experience, which may have provided important context for interpreting differences in consent representations. Fourth, the lack of qualitative data limits the ability to capture the nuanced, context-dependent nature of how adolescents interpret and negotiate sexual consent. Fifth, the questionnaire was initially developed as part of a broader study on mental health. As a result, the sample may overrepresent adolescents with a particular interest in or experience of mental health issues, which may limit the generalisability of the findings. Sixth, dissemination of the survey via social media likely excluded adolescents who are not engaged on these platforms, potentially resulting in a coverage bias.

Future research should adopt longitudinal models to examine how adolescents’ perceptions of consent evolve with age, biopsychosocial development and gain experience and knowledge in sexual health. Future research would also benefit from probabilistic sampling strategies and the integration of qualitative methods to provide a more comprehensive understanding of the perception of sexual consent.

It would be valuable to examine the effectiveness of specific educational interventions in shifting adolescents’ understanding of sexual consent and their behaviours in sexual relationships. Assessing the efficacy of various educational interventions—including distinct target groups (eg, individuals, educators, healthcare providers, policymakers), delivery methods (eg, classroom, community, digital) and content focus (eg, knowledge, attitudes, communication skills)—is crucial.^40^

Ensuring that all adolescents receive equitable, developmentally appropriate education on sexual consent is not only a matter of public health and safety but also a fundamental step toward fostering respect and autonomy in all adolescent relationships.^41^

## Data Availability

Data are available on reasonable request.
