# Supplementary material for: Youth characteristics in relation to their perceptions of sexual consent: a cross-sectional study
Source: BMJ Paediatr Open. 2026 Jun 22;10(1):e004157. doi: 10.1136/bmjpo-2025-004157 (PMC13289400; doi:10.1136/bmjpo-2025-004157)
Supplement: Supplementary file 1 [file bmjpo-2025-004157.reviewer_comments.pdf]

PEER REVIEW HISTORY

BMJ Paediatrics Open publishes all reviews undertaken for accepted manuscripts. Reviewers are asked to complete a checklist review form and are provided with free text boxes to elaborate on their assessment. These free text comments are reproduced below.

ARTICLE DETAILS

**TITLE (PROVISIONAL)**	Youth Characteristics in Relation to their Perceptions of Sexual Consent: a cross-sectional study
**AUTHORS**	Knob, Cyril<br>Chok, Lorraine<br>Delacrétaz, Romaine<br>Barrense-Dias, Yara<br>Ambresin, Anne- Emmanuelle

VERSION 1 – REVIEW

**REVIEWER NAME**	**Colombo, Annamaria**
**REVIEWER AFFILIATION**	Haute ecole de travail social Fribourg
**REVIEWER CONFLICT OF INTEREST**	No Conflict of Interest
**AI DISCLOSURE**	I have used AI to help me for the translation in English.
**DATE REVIEW RETURNED**	12-Nov-2025

**GENERAL COMMENTS**	The article is well written, clearly structured, and makes a relevant contribution to a topic that is both socially and scientifically topical. As I am not specialized in quantitative methodologies, I cannot comment on the quantitative analyses carried out. The literature review is short but appropriate and based on recent references. However, the concept of sexual consent, which is widely debated in the literature, seems to me to be insufficiently discussed on a theoretical level. The affirmative consent approach is defined, but in my view it is not sufficiently contextualized in relation to the well-documented difficulty in the literature of clearly defining consent and the different approaches to this concept according to discipline. Furthermore, the social, cultural, and psychological barriers that often prevent the implementation of true affirmative consent, particularly for women and sexual and emotional minorities, are not mentioned at all.<br>As a result, the three categories used for the analysis, "Affirmative consent perception," "Mixed perception," and "Misaligned perception," are unconvincing. I question in particular the choice of the term "misaligned," which could suggest a value judgment. In the absence of prior theoretical discussion, the construction of this analytical framework may suggest that only the affirmative consent approach is "right," with the others not being "correct" interpretations. Rather than a "right"/"wrong" type categorization, wouldn't it be more appropriate to use a typology that reflects different ways of representing sexual consent?<br>Furthermore, if I understand correctly, these representations of consent are based on the responses to the statements proposed in the questionnaire and found in Table 2. However, these statements are not discussed in the article, and the extent to which they are or are not representative of an affirmative consent representation remains implicit. Furthermore, in my opinion, the scoring system is not explained sufficiently and remains unclear. The sentence "any response that did not align with the expected response for affirmative and active sexual consent were scored as 1 (vs. 0)" contains implicits that, in my view, should be clearly explained: what are the "expected responses for affirmative and active sexual

	consent"? What does a response that "did not align" with these expectations mean in concrete terms?<br><br>The discussion of the results is interesting and makes a real contribution to our understanding of the links between young people's perceptions of consent, gender, age, and social background, as well as suggesting ways to better support these young people and prevent sexual violence by better targeting prevention efforts according to their socio-demographic characteristics. However, it would gain in depth and relevance if the definition of consent were more nuanced and the categories were better grounded theoretically, as suggested above.

**REVIEWER NAME**	Moussaoui, Dehlia
**REVIEWER AFFILIATION**	
**REVIEWER CONFLICT OF INTEREST**	No Conflict of Interest
**AI DISCLOSURE**	No, I have not used AI
**DATE REVIEW RETURNED**	02-Apr-2026

**GENERAL COMMENTS**	Thank you for the opportunity to review this manuscript, which addresses an important topic. The study appears methodologically sound, the results are relevant and the conclusions are supported by the data. There are some inconsistencies in how the results are reported. I believe the paper would be suitable for publication after revision.<br><br>Major comments<br><br>Introduction:<br>1. I suggest reshaping and shortening the Introduction, to provide only the essential background and clearly identify the gap in current knowledge, thereby making a more compelling case for the importance of this study. I recommend mentioning the terms “positive sexuality” or “comprehensive sexuality education” when discussing the promotion of sexual health. The section from page 4 line 30 (“Previous studies have shown…”) to page 5 line 10 (“… the consumption of alcohol or other substances”) should be synthesized.<br><br>Methods:<br>2. Was information available regarding the sexual activity or experience of the participants? If so, it should be described.<br>3. Page 6, line 31: Who developed these statements on sexual consent and how were they chosen? Similarly, who developed the scoring system and how was it developed (page 7, line 48)? The sentence on page 8 line 19-23 is unclear (“The cut-off of 3 or more was chosen…”).<br><br>Results:<br>4. There are some inconsistencies in how the results are reported. They should be presented in a complete and consistent manner, as on page 10, line 46-53:<br>- Page 9 line 38-49 and page 10 line 3-12: statistical test results should be reported for all findings in brackets (and not only the proportions and/or p-value)<br>- Page 10 line 17-44: please provide the p-value in addition to the results in brackets.<br><br>Discussion:<br>5. Page 12, line 28-40: I suggest providing some context on how sex education is delivered in Switzerland, including the age at which it is

	introduced, to help the reader understand and contextualize the findings about younger age/engagement in apprenticeships/etc. and a higher risk of misaligned perception of sexual consent.<br><br>Minor comments<br><br>Methods:<br>6. Page 6, line 44: please remove age in brackets (14-19 years old)<br><br>Results:<br>7. Table 1:<br>- Rows: Please provide all data including "No" responses (e.g. Victim of sexual assault, Physical or verbal violence from partner, etc.). Were they any missing data? If so, this should be mentioned.<br>- Columns: The "mean+/-SE" doesn't seem to correspond to any presented data. Please remove.<br>8. Table 2: please provide both absolute numbers and percentages. Statistical test used for the analyses should be mentioned in the legend.<br>9. Table 3: Sex should be replaced by Gender; I suspect that a second asterisk is missing next to the "other" category (2.8%). The statistical tests used for the analyses should be mentioned in the legend.<br>10. Table 4: Sex should be replaced by Gender.<br>11. Figure 1 appears twice in the document available to me. Please remove one of them. Eleven participants were excluded because they "did not give honest answers": how was the honesty of the responses evaluated? Did the authors mean "consistent answers"? How was this assessed?<br><br>Discussion:<br>12. Page 11, line 8 and 19: While I strongly recommend reporting results in both absolute numbers and percentage in the Results section and Tables, this is not necessary in the Discussion, where the presentation can be simplified (report only percentages or approximates "just above half….").<br>13. Page 14, line 3-5: "…but also to the broader goal of supporting positive youth development and well-being" please add "positive sex education" or "positive sexuality".<br>14. Page 14, line 26: please replace sex by gender<br>15. Fifth and sixth limitations: please clarify that these may limit the generalizability of the findings.

VERSION 1 – AUTHOR RESPONSE

Reviewer: 1

Annamaria Colombo, Haute ecole de travail social Fribourg Comments to the Author

The article is well written, clearly structured, and makes a relevant contribution to a topic that is both socially and scientifically topical. As I am not specialized in quantitative methodologies, I cannot comment on the quantitative analyses carried out. The literature review is short but appropriate and based on recent references.

However, the concept of sexual consent, which is widely debated in the literature, seems to me to be insufficiently discussed on a theoretical level. The affirmative consent approach is defined, but in my view it is not sufficiently contextualized in relation to the well-documented difficulty in the literature of clearly defining consent and the different approaches to this concept according to discipline. Furthermore, the social, cultural, and psychological barriers that often prevent the implementation of true affirmative consent, particularly for women and sexual and emotional minorities, are not mentioned at all.

Thank you for this observation, we agree that stronger theoretical grounding would be better. We made

revisions on that point in two ways. First, we now introduce three broad conceptual approaches to sexual consent documented in the literature - implicit, affirmative, and contextual/relational models (Muehlenhard et al., 2016; Jozkowski et al., 2014; Willis et al., 2018; Anyadike-Danes et al., 2024). This provides the theoretical context within which our three empirical categories can be interpreted, and directly addresses the concern that the categorization framework may appear to imply a simple right/wrong binary. Second, we have added a sentence explicitly acknowledging the social, cultural, and psychological barriers constraining the enactment of affirmative consent - particularly for women and young people from sexual and gender minorities.

As a result, the three categories used for the analysis, "Affirmative consent perception," "Mixed perception," and "Misaligned perception," are unconvincing. I question in particular the choice of the term "misaligned," which could suggest a value judgment. In the absence of prior theoretical discussion, the construction of this analytical framework may suggest that only the affirmative consent approach is "right," with the others not being "correct" interpretations. Rather than a "right"/"wrong" type categorization, wouldn't it be more appropriate to use a typology that reffects different ways of representing sexual consent?

We agree. Building on the theoretical framework introduced in the revised Introduction (see response above), the three categories have been renamed to reflect a continuum of consent representations rather than a normative hierarchy:

- **"A=irmative"** perception group : consistent with an explicit and active consent model
- **"Hybrid"** perception group: endorses affirmative consent in principle but retains some reliance on contextual or implicit cues, reflecting the tension between endorsed norms and real-life scripts documented in the literature
- **"Implicit"** perception group: consistent with a presumptive or non-refusal consent model, in which consent is inferred from context, appearance, or the absence of resistance

This reframing positions the three profiles as empirically derived representations along a continuum of consent representations rather than as a judgment of correctness. The term "misaligned" has been removed throughout the manuscript.

Furthermore, if I understand correctly, these representations of consent are based on the responses to the statements proposed in the questionnaire and found in Table 2. However, these statements are not discussed in the article, and the extent to which they are or are not representative of an affirmative consent representation remains

implicit. Furthermore, in my opinion, the scoring system is not explained sufficiently and remains unclear. The sentence "any response that did not align with the expected response for affirmative and active sexual consent were scored as 1 (vs. 0)" contains implicits that, in my view, should be clearly explained: what are the "expected responses for affirmative and active sexual consent"? What does a response that "did not align" with these expectations mean in concrete terms?

The Methods section has been substantially revised to address these concerns. First, the origin and theoretical grounding of the statements are now explicitly stated: the eight statements were developed by the authors, drawing on the principles underlying recent Swiss legal reforms and the sexual consent literature, according to which affirmative consent must be actively expressed, freely given, clear, and cannot be inferred from silence, passivity, or contextual cues. Second, the scoring logic is now explicitly stated: responses inconsistent with the affirmative consent standard - including "did not know" answers - were scored 1 (vs. 0 for consistent responses), removing any ambiguity about what "did not align" means in concrete terms.
Specifically, disagreement with statement 1 and agreement with statements 2 through 7 were coded as inconsistent with this standard, reflecting whether each response endorsed or contradicted the principle that consent must be actively expressed. Third, the cut-off of 3 or more for the implicit perception group is now justified on the overall distribution of scores in the sample, reflecting a pattern of responses predominantly inconsistent with affirmative consent principles across the majority of statements.

The discussion of the results is interesting and makes a real contribution to our understanding of the links between young people's perceptions of consent, gender, age, and social background, as well as suggesting ways to better support these young people and prevent sexual violence by better targeting prevention efforts according to their socio-demographic characteristics. However, it would gain in depth and relevance if the definition of consent were more nuanced and the categories were better grounded theoretically, as suggested above

Thank you for this encouraging assessment. As detailed in our responses above, the theoretical framework has been substantially strengthened in the Introduction and Methods, and the Discussion language has been revised accordingly to ensure consistency with the new theoretical framing throughout. Together, these revisions provide the theoretical depth identified as lacking, and we believe they strengthen the Discussion by allowing the findings to be interpreted within a more robust conceptual framework.

Reviewer: 2 Dehlia Moussaoui

Comments to the Author

Thank you for the opportunity to review this manuscript, which addresses an important topic. The study appears methodologically sound, the results are relevant and the conclusions are supported by the data. There are some inconsistencies in how the results are reported. I believe the paper would be suitable for publication after revision.

Major comments

Introduction:

1. *I suggest reshaping and shortening the Introduction, to provide only the essential background and clearly identify the gap in current knowledge, thereby making a more compelling case for the importance of this study. I recommend mentioning the terms "positive sexuality" or "comprehensive sexuality education" when discussing the promotion of sexual health. The section from page 4 line 30 ("Previous studies have shown…") to page 5 line 10 ("… the consumption of alcohol or other substances") should be synthesized.*

Section from "Previous studies have shown…" to "…consumption of alcohol or other substances" were synthesized into a single focused paragraph. We note, however, that the Introduction has been partially also reshaped in response to Reviewer 1's suggestion for stronger theoretical grounding - specifically through the integration of three broad conceptual models of consent and the barriers constraining affirmative consent in practice. The net result is an Introduction that is more concise in its empirical review while being more substantive in its theoretical framing. Additionally, the terms "positive sexuality" and "comprehensive sexuality education" have been integrated into the paragraph discussing health promotion.

Methods:

2. Was information available regarding the sexual activity or experience of the participants? If so, it should be described.

Unfortunately, no information on participants' sexual activity or experience was collected in the original survey. We agree with the reviewer that this is a relevant variable that could have provided important context for interpreting consent representations; in particular, prior sexual experience has been shown in litterature to influence consent communication and understanding. We have therefore added this to the limitations of the study.

3. Page 6, line 31: Who developed these statements on sexual consent and how were they chosen? Similarly, who developed the scoring system and how was it developed (page 7, line 48)? The sentence on page 8 line 19-23 is unclear ("The cut-off of 3 or more was chosen…").

As detailed in the response to Reviewer 1 above, the Methods section has been revised to explicitly state that the statements and scoring system were developed by the authors, grounded in the affirmative consent literature and Swiss legal reform principles. The cut-off sentence has been rewritten.

Results:

4. There are some inconsistencies in how the results are reported. They should be presented in a complete and consistent manner, as on page 10, line 46-53:

- Page 9 line 38-49 and page 10 line 3-12: statistical test results should be reported for all findings in brackets (and not only the proportions and/or p-value)

- Page 10 line 17-44: please provide the p-value in addition to the results in brackets. The Results section has been revised to ensure complete and consistent reporting throughout: complete test results inclunding p-values have been added to all findings, in the bivariate and multivariable paragraphs, following the format already used for the regression results. We hope this revision adequately addresses the reviewer's concern, and would be grateful for any further clarification; should additional adjustments be requiered.

Discussion:

5. Page 12, line 28-40: I suggest providing some context on how sex education is delivered in Switzerland, including the age at which it is introduced, to help the reader understand and contextualize the findings about younger age/engagement in apprenticeships/etc. and a higher risk of misaligned perception of sexual consent.
A sentence has been added to the Discussion, drawing on existing Swiss data [Barrense-Dias et al., 2020]. This describes variability in quality and coverage across linguistic regions, and the discontinuation of structured sex education for young people who leave mandatory schooling as early as age 15–16 to enter vocational trainin; providing direct context for the findings on age and apprenticeship status as risk factors for an implicit consent perception.

Minor comments

Methods:

6. Page 6, line 44: please remove age in brackets (14-19 years old) Removed

Results:

7. Table 1:

- Rows: Please provide all data including "No" responses (e.g. Victim of sexual assault, Physical or verbal violence from partner, etc.). Were they any missing data? If so, this should be mentioned.

- Columns: The "mean+/-SE" doesn't seem to correspond to any presented data. Please remove.

We considered adding "No" responses for all binary variables; however, since these correspond strictly to the complement of the reported proportions (i.e. 100% minus the reported percentage), including them would not add information while substantially increasing the size of the table. A note has therefore been added to the legend to clarify that for binary variables, only the proportion of positive responses is reported.
No missing data were identified in the dataset, which is now explicitly stated in the Methods section.
The "mean±SE" column header, has been removed.

8. Table 2: please provide both absolute numbers and percentages. Statistical test used for the analyses should be mentioned in the legend.
Given that all analyses were conducted on a weighted sample, reporting absolute numbers alongside percentages could be misleading, as weighted counts are not whole numbers. We have therefore opted to report percentages only, and a note has been added to the legend clarifying that all percentages reflect weighted sample estimates.
The legend has also been updated to specify that chi-square tests were used for all comparisons.

9. Table 3: Sex should be replaced by Gender; Done. I suspect that a second asterisk is missing next to the "other" category (2.8%). Indeed, added. The statistical tests used for the analyses should be mentioned in the legend. Done. The legend of Table 3 has been updated to specify that chi-square tests were used for categorical variables and ANOVA for continuous variables.

10. Table 4: Sex should be replaced by Gender. Done.

11. Figure 1 appears twice in the document available to me. Please remove one of them. Eleven participants were excluded because they "did not give honest answers": how was the honesty of the responses evaluated? Did the authors mean "consistent answers"? How was this assessed?
Regarding the duplicate Figure 1, we apologize for this inconvenience, which is likely an artifact of the submission platform; we would kindly ask the editors to confirm and remove the duplicate if necessary.
Regarding the eleven excluded participants, a closing question asked participants whether their answers were sufficiently honest to be used for research purposes - a self-reported data quality check previously validated in adolescent surveys addressing

sensitive topics [Siegel et al., 1998, Narring et al.,2002]. Participants responding negatively were excluded. This is now stated explicitly in the Methods.

Discussion:

12. Page 11, line 8 and 19: While I strongly recommend reporting results in both absolute numbers and percentage in the Results section and Tables, this is not necessary in the Discussion, where the presentation can be simplified (report only percentages or approximates "just above half….").
Done. Absolute numbers have been removed throughout the Discussion, with results now reported as percentages or approximations where appropriate.
13. Page 14, line 3-5: "…but also to the broader goal of supporting positive youth development and well-being" please add "positive sex education" or "positive sexuality".
"Positive sexuality" added.
14. Page 14, line 26: please replace sex by gender Done.
15. Fifth and sixth limitations: please clarify that these may limit the generalizability of the findings.
Done. Generalizability statements have been added to both the fifth and sixth limitations.

Additional correction

In the process of revising the manuscript, a typographical error in the sample size reported in the text and flowchart (N=998 instead of N=988) has been corrected. All statistical analyses were conducted on the correct sample of N=988, as reflected in the tables, and this error has no bearing on any of the reported results.

VERSION 2 – REVIEW

**REVIEWER NAME**	Colombo, Annamaria
**REVIEWER AFFILIATION**	Haute ecole de travail social Fribourg
**REVIEWER CONFLICT OF INTEREST**	No Conflict of Interest
**AI DISCLOSURE**	Deepl for translation in English
**DATE REVIEW RETURNED**	29-May-2026

**GENERAL COMMENTS**	The authors have made substantial revisions to the article. The three conceptual models (implicit, affirmative, contextual/relational) are now more clearly highlighted and discussed, as are the social, cultural and psychological barriers to affirmative consent. Methodologically, this new version provides greater clarity regarding the origin of the items, the coding direction (disagreement with item 1, agreement with items 2–7 = inconsistent), and the justification for the threshold of ≥3. The decision to rename the groups 'Affirmative / Hybrid / Implicit' perception of consent strikes me as convincing, much clearer and less normative. The discussion has been improved, particularly by drawing on the new theoretical framework, but it remains relatively brief on the conceptual complexity of consent. Nevertheless, it seems to me that this article can now be published with the amendments made.

**REVIEWER NAME**	**Moussaoui, Dehlia**
**REVIEWER AFFILIATION**	
**REVIEWER CONFLICT OF INTEREST**	No Conflict of Interest
**AI DISCLOSURE**	No, I have not used AI
**DATE REVIEW RETURNED**	30-May-2026

**GENERAL COMMENTS**	I am satisfied with the authors' revisions and their answers to the comments. I believe the manuscript is now suitable for publication.
